# When ecological transitions are not so infrequent: independent colonizations of athalassohaline water bodies by Arcellidae (Arcellinida; Amoebozoa), with descriptions of four new species

**DOI:** 10.1093/femsec/fiad076

**Published:** 2023-07-06

**Authors:** Fernando Useros, Rubén González-Miguéns, Carmen Soler-Zamora, Enrique Lara

**Affiliations:** Real Jardín Botánico (RJB-CSIC), Department of Mycology, Moyano 1, Plaza Murillo 2, 28014 Madrid, Spain; Real Jardín Botánico (RJB-CSIC), Department of Mycology, Moyano 1, Plaza Murillo 2, 28014 Madrid, Spain; Real Jardín Botánico (RJB-CSIC), Department of Mycology, Moyano 1, Plaza Murillo 2, 28014 Madrid, Spain; Real Jardín Botánico (RJB-CSIC), Department of Mycology, Moyano 1, Plaza Murillo 2, 28014 Madrid, Spain

**Keywords:** Arcellinida, athalassohaline, ecological transition, euryhaline, salinity barrier, testate amoebae

## Abstract

The salinity and humidity barriers divide biodiversity and strongly influence the distribution of organisms. Crossing them opens the possibility for organisms to colonize new niches and diversify, but requires profound physiological adaptations and is supposed to happen rarely in evolutionary history. We tested the relative importance of each ecological barrier by building the phylogeny, based on mitochondrial cytochrome oxidase gene (COI) sequences, of a group of microorganisms common in freshwater and soils, the Arcellidae (Arcellinida; Amoebozoa). We explored the biodiversity of this family in the sediments of athalassohaline water bodies (i.e. of fluctuating salinity that have non-marine origins). We found three new aquatic species, which represent, to the best of our knowledge, the first reports of Arcellinida in these salt-impacted ecosystems, plus a fourth terrestrial one in bryophytes. Culturing experiments performed on *Arcella euryhalina* sp. nov. showed similar growth curves in pure freshwater and under 20 g/L salinity, and long-term survival at 50 g/L, displaying a halotolerant biology. Phylogenetic analyses showed that all three new athalassohaline species represent independent transition events through the salinity barrier by freshwater ancestor, in contrast to the terrestrial species, which are monophyletic and represent a unique ecological transition from freshwater to soil environments.

## Teaser

Arcellidae, a mainly freshwater testate amoebae family, has crossed the salinity barrier multiple times to non-marine saline ecosystems, but has had only one (ancient) transition to terrestrial ecosystems.

## Introduction

The distribution of biodiversity at a global scale is structured by the major ecosystems, which are delimited by ecological barriers (Simpson 1944). Among these, the salinity barrier, which separates marine and continental saline systems from other environments, is by far the best studied. It divides biodiversity at a global scale, because crossing it demands profound physiological and metabolic adaptations. The efficiency of this barrier has been well documented in animals, whose internal organs are separated from the environment by skin or an exoskeleton, which helps to regulate osmotic processes (Hutchinson 1975). Unicellular eukaryotes (protists), however, are more permeable and require specialized organelles (i.e. contractile vacuoles) (Docampo et al. 2013) and/or metabolic pathways that enable osmoregulation (i.e. ionic pumps and transporters or compatible solutes) (Harding et al. 2017).

Ecological transitions between these ecosystems have been documented in several clades (Lee and Bell 1999), but are considered to happen rarely in the evolutionary history of eukaryotes, microbial or not (Hutchinson 1975, Logares et al. 2009). Still, along with an increasing knowledge of protist systematics, a growing number of new instances of transitions through the salinity barrier are appearing, as in cryptophytes (Filker et al. 2017), diatoms (Alverson et al. 2007, Roberts et al. 2022), dinoflagellates (Annenkova et al. 2020) or testate amoebae (González-Miguéns, Soler-Zamora, Fernando Useros et al. 2022, González-Miguéns et al. 2023).

The salinity barrier has been traditionally studied in marine groups that subsequently adapted to freshwater (Lee and Bell 1999). Although less frequent and less studied, there are cases in the opposite direction, such as the marine Trichoptera family Chathamidae (Riek 1977). Nevertheless, these studies are generally conducted in brackish environments with a clear connectivity between marine and freshwater. In this case, our aim was to study the transitions of a primarily freshwater group to saline and marine systems. For this reason, our study took place in athalassohaline systems, which, by definition, are those saline systems that have never been connected to the sea and whose salinity level and composition are different from seawater (Last 2002, Oren 2007, Safarpour et al. 2018). They typically occur in endorheic drainage basins under arid or semiarid climates where evaporation exceeds precipitation (Hammer 1986, O'leary and Glenn 1994, Wurtsbaugh et al. 2017). Environmental parameters in these lakes often vary to extreme values. Salinity can fluctuate along the year and reach values much higher than the average 35 g/L of seawater. Temperatures and solar radiation are usually elevated and water levels fluctuate greatly. Like in estuaries, the organisms that inhabit athalassohaline systems need important physiological adaptations to cope with these variations (Mesbah and Wiegel 2011, Plemenitaš and Gunde-Cimerman 2011, Harding et al. 2017). All these characteristics and their lack of connection with the marine environment make them an interesting case, ideal for the study of ecological transitions from freshwater to saline environments.

Similarly to the salinity barrier, the humidity barrier, which we define here as the separation between terrestrial and aquatic environments, also demands profound adaptations to withstand the constraints of living out of water, that is, desiccation, or underwater, that is, osmotic pressure and gas exchange. The humidity barrier has structured much of vascular plants' evolutionary history and generated changes in their anatomy, physiology and reproductive biology (Kenrick and Crane 1997, Niklas 2000). In animals, the most obvious examples are the colonization of land by amphibians (Schoch 2014) and the ancestor of insects (Grimaldi et al. 2005, Rota-Stabelli et al. 2013). Transitions from terrestrial to aquatic systems can be witnessed in the evolutionary history of marine mammals (Fordyce and Barnes 1994) and also in many insect groups, such as Gyrinidae, Nepidae, Odonata, Trichoptera or Ephemeroptera (Grimaldi et al. 2005). In protists, the colonization of terrestrial environments requires the capacity for forming desiccation-resistant structures (cysts, spores or sclerotia) (Geisen et al. 2018). These structures are widespread in the tree of eukaryotes, and although they have not been studied in depth in free-living forms, they are likely to not be homologous in all groups and to have been ‘reinvented’ independently in many clades (Geisen et al. 2018). The humidity barrier has been relatively less studied in protists than the salinity barrier (Geisen et al. 2017). A recent survey of protists global diversity in soil, freshwater and marine systems suggests nevertheless that aquatic (marine and freshwater) and terrestrial communities do differ (Singer et al. 2021), which shows that the humidity barrier is effective in protists. However, which barrier is the most difficult to overcome for unicellular eukaryotes still remains to be determined.

In order to evaluate the relative importance of salinity and humidity in the evolutionary history of other eukaryotic microorganisms, we took the family Arcellidae (Sphaerothecina; Arcellinida; Elardia; Amoebozoa) as a study group. The order Arcellinida, to which Arcellidae belong, occurs worldwide in different terrestrial, wetland and freshwater habitats (Smith et al. 2007), with very few species recorded in coastal salt marshes and brackish waters (Golemansky 1998, Charman et al. 2002, Gehrels et al. 2006, González-Miguéns et al. 2023). They are often ecological specialists with narrow tolerance to alterations of their environment (Singer et al. 2018), hence their use as bioindicators for environmental health (Roe and Patterson 2014, Creevy et al. 2018, Kosakyan and Lara 2019). The family Arcellidae are not typically found in saline environments, although they have been episodically found in coastal marshes (Golemansky 1991, 1998, Charman et al. 2002, Gehrels et al. 2006), anchialine cenotes with subterranean seawater connections (van Hengstum et al. 2008) and coastal peatlands exposed to salt spray (Whittle et al. 2018). While the original habitat of the common ancestor of all Arcellinida remains unclear, family Arcellidae most probably appeared in aquatic habitats (González-Miguéns, Todorov et al. 2022). Indeed, only certain derived species from genus *Galeripora*, such as those from the *G. arenaria* complex (González-Miguéns, Soler-Zamora, Villar-Depablo et al. 2022), are strictly terrestrial and can settle down even in arid environments, while stem groups are aquatic.

In this work, we trace the ancestral habitat of the family Arcellidae in order to infer the relative importance of these ecological barriers in the evolution of this family. First, we isolated single cells from different athalassohaline lakes, streams and surrounding soils ecosystems in central Spain. Then we inferred the ancestral habitat of the different lineages in the family Arcellidae (marine/freshwater/soil/*Sphagnum*) based on ancestral trait reconstruction to evaluate the relative frequency of ecological transitions. Moreover, we inferred the salinity tolerance (euryhaline vs. stenohaline) for one species using a culturing approach to measure growth rates under different salt concentrations. Finally, we formally described the new species encountered.

## Materials and Methods

### Study area

Spain, due to its geomorphology and its generally dry climate, has several important athalassohaline systems distributed in small-size patches in different endorheic basins (Pardo 1948, Comin and Alonso 1988). Samples for this study were collected from two main sites: Salobral de Ocaña (39º 59' N, 3º 36' W) and the ‘Special Area of Conservation’ of Petrola, Salobrejo and Corral Rubio saline lakes (38º 50' N, 1º 33' W).

Salobral de Ocaña is a salt marsh located on a gypsum-rich substrate crossed by several streams and temporary lakes (Angeler et al. 2006, Menéndez Pidal et al. 2021). Salt composition consists mainly of SO_4_^2−^-Ca^2+^-Na^+^ (Menéndez Pidal et al. 2021). We sampled the top millimeters of sediment at different points along three streams that eventually joined into a single one during February and September 2020 and January 2021.

The Special Area of Conservation of Petrola, Salobrejo and Corral Rubio salt lakes, included in the Red Natura 2000, is a wetland formed by more than 20 endorheic lakes, most of them temporary (Donate et al. 2004, Gómez-Alday et al. 2014). Lake Petrola is the largest and most permanent, as well as the best studied. Its ion composition is Mg^2+^-Na^+^-Cl^−^-SO4^2−^ during summer and fall, and changes to Mg^2+^-Cl^−^-SO4^2-^ in early spring (Ordóñez et al. 1973, Valiente et al. 2017). The lakes suffer several anthropogenic pressures due to agriculture and wastewater disposal (Gómez-Alday et al. 2014). We collected several sediment samples at the borders of the lakes of Horna, Petrola and Corral Rubio's Large lake during February and October 2020 and March 2021.

### Microscopic observations

Samples were transferred to a Petri dish to be observed under an inverted microscope (Leica DMI8), up to 400x magnification DIC. A Leica MC170 HD camera with the Leica application suite (v. 4.12.0) software was used to take photographs.

Observed individuals were isolated using a small diameter pipette and transferred to a drop of sterile water, then further rinsed into another drop in order to get rid of other eukaryotic contaminants. Living cells were stored, individually or in groups of up to four cells, in Eppendorf tubes containing 100 µL of guanidine thiocyanate-based nucleic acids extraction buffer (Chomczynski and Sacchi 1987) for later DNA extraction.

Some individuals or empty tests were deposited, after washing in distilled water, on stubs for scanning electron microscopy. Stubs were desiccated in a box with silica gel at least one day before metallization and observation. Then they were coated with 8-nm gold using a Balzers SCD 004 sputter coater and a tension of 15 kV. They were observed with a Hitachi S-3000 N and a JEOL JSM-5510 (operating at 10 kV) scanning electron microscope.

### DNA extraction and amplification

Single cell DNA extractions were performed on cells stored in guanidine thiocyanate buffer, as described in Duckert et al. (2018).

We amplified the COI region using the semi-nested protocol described in González-Miguéns, Soler-Zamora, Villar-Depablo et al. (2022); a first amplification was performed using the mitochondrial cytochrome c oxidase subunit I universal primer pair LCO 1490 (5′ GGTCAACAAATCATAAAGATATTGG 3′) and HCO 2198 (5′ TAAACTTCAGGGTGACCAAAAAATCA 3′) (Folmer et al. 1994) with the following PCR program: initial denaturation at 96°C for 5 min, followed by 40 cycles at 94°C for 15 s, 40°C for 15 s and 72°C for 90 s and a final extension step at 72°C for 10 min. We used the product of this first PCR, usually diluted 1:20, as the base for a second amplification using the Arcellinida-specific primers ArCOIF (5′ GGTATTYTAGCWCATTCNRGTGG 3′) (González-Miguéns, Soler-Zamora, Villar-Depablo et al. 2022) coupled with HCO, and its reverse and complementary ArCOIR coupled with LCO. The PCR profile for this step was an initial denaturation at 96°C for 5 min, followed by 35 or 40 cycles at 94°C for 15 s, 55°C for 15 s and 72°C for 90 s, and a final extension step at 72°C for 10 min (González-Miguéns, Soler-Zamora, Villar-Depablo et al. 2022). After each amplification, 3 μL of the reaction was analyzed by electrophoresis on a 1% agarose gel to verify fragment size and check for contaminations. Final products with bands of expected size were run on a 1% agarose gel and the bands were excised and stored at 4ºC or −20ºC. The samples were sequenced using Sanger dideoxy-technology in both directions by Macrogen Inc. (Macrogen Europe, Madrid, Spain). Control quality of the raw sequences and assembling of both PCR products were carried out using the software GENEIOUS PRIME (v. 2019.0.4). Finally, we performed a blastn analysis (Altschul et al. 1990) against the GenBank database to ensure that our sequences belonged to Arcellinida.

### Phylogenetic and ancestral habitat reconstruction analyses

We aligned our sequences with other Arcellidae sequences present in Genbank, as well as some Netzeliidae and Hyalospheniformes as outgroups. We also included Arcellidae environmental sequences from González-Miguéns et al. (2023), altogether resulting in a total of 76 sequences. A first alignment was performed using the MAFFT algorithm (Katoh et al. 2002), as implemented in GENEIOUS PRIME (v. 2019.0.4). This alignment was manually edited using Aliview (v. 1.27) (Larsson 2014). Phylogenetic trees were obtained using maximum likelihood and Bayesian inferences.

Maximum likelihood analyses were performed using the online version of IQ-TREE (Nguyen et al. 2015, Trifinopoulos et al. 2016), allowing it to search for the best substitution model with free rate heterogeneity; 10'000 non-parametric bootstraps were performed to assess node supports.

Bayesian inference phylogenetic analysis was performed using MrBayes 3.2.7a (Huelsenbeck and Ronquist 2001) as implemented in CIPRES (Miller et al. 2011). We used the substitution parameters obtained in IQ-TREE as priors for the analysis. We performed two independent runs consisting of four MCMC chains of 10^7^ generations. Trees were sampled every 1000 generations, and the first 25% were discarded as burn-in.

We inferred the habitat of the ancestors of each clade by phylogenetic ancestral character reconstruction. We considered four habitats: freshwater, saline water, terrestrial and *Sphagnum* (peat bog). Because it creates permanently wet microhabitats for associated microbes (coined the *sphagnosphere*, Jassey et al. 2011), *Sphagnum* was considered here as akin to aquatic (freshwater). We used the R package phytools (Revell 2012) to trim the tree and join it to the database with the habitat trait. We fitted evolutionary models with equal, symmetric or all-different transition rates using the *fitDiscrete* function of the package geiger v. 2.0.10 (Pennell et al. 2014). We used the equal-rates model, because it had the lowest Akaike's information criterion, for the ancestral character estimation, using the *ace* function in the ape v. 5.6.2 package (Paradis and Schliep 2019).

### Comparing growth at different salinities

Organisms from a strain superficially looking like *Arcella intermedia* were isolated in Petri dishes for the purpose of cultivation. We used autoclaved water initially collected from a highly saline stream situated in the Salobral de Ocaña (with a salinity of 75 g/L) as a medium that we diluted to reach the desired salinities. We also added autoclaved rice grains as the nutrient source for the environmental bacteria that would be co-inoculated with the amoebae, and that would serve as prey. Cultures were stored at 15ºC.

The cultures were set at different salinities: freshwater (autoclaved), 20 g/L (the salinity of the sample from which individuals were isolated) and 50 g/L (the maximum salinity measured in the main body of Pétrola lake). Eight cells per culture were deposited in each Petri dish; we had eight culture replicates for treatments of 0 and 20 g/L, respectively, and four for the 50 g/L treatment. We then counted the number of living cells and dead individuals every 2–3 days during 20 days, in order to obtain a growth curve for each treatment.

Statistical analyses were performed using R software v. 4.1.3 (R Core Team 2022) implemented in R studio v. 1.3.1093 (RStudio Team 2020). In order to test how far growth curves diverged from each other or not, we adjusted Poisson and quasi-Poisson general linear models using the glm function of the stats base R package and a negative binomial model using the glm.nb function of the MASS package (Venables and Ripley 2002). We tested for overdispersion using the gof function of the aods3 package (Lesnoff and Lancelot 2022) and selected the model that adjusted better. We checked for significant differences between the treatments using ANOVA type III as implemented in the package car (Fox and Weisberg 2019).

### Morphometric analysis

We took the following measurements: shell diameter, aperture diameter and preapertural area, and averaged them. When possible, we also measured the height of the test and the preapertural depth. We also included in our dataset the measurements of shell and aperture diameter from González-Miguéns, Soler-Zamora, Villar-Depablo et al. (2022), in order to detect possible similarities or differences with those other species of Arcellidae.

Statistical analyses were performed using R software v. 4.1.3 (R Core Team 2022) implemented in R studio v. 1.3.1093 (RStudio Team 2020). We plotted the distribution of each variable by morphospecies and sample location to check for different clusters.

We also performed a PCA, using the core package stats, for this purpose. We also conducted linear discriminant analysis (LDA) using the package MASS v. 7.3.55 (Venables and Ripley 2002), which identifies the combination of morphological variables that could be used to delimitate the clades. We used 75% of the dataset as a training set for the LDA, and 25% as a testing set. The ggplot2 package (Wickham et al. 2016) was used to graphically represent the results.

## Results

### Abiotic parameters on the sampling sites

We observed large fluctuations in salinity between different sampling points at the same site and between different sampling times (i.e. in Pétrola lake, salinities ranged from 2.7 to 37 g/L in March, but in October they increased from 48 to 142 g/L). The total salinity ranges of each locality are shown in Table 1 (more data are provided in the Supplementary material). The salinity values at which living specimens of each species were found are indicated in Table 2.

**Table 1.** Salinity and pH range of the samples at each of the sites.

Site	Salinity (g/L)	pH	Coordinates
Salobral de Ocaña	6–126.7	NA	39º 59′ N, 3º 37′ W
Horna lake	4.7–15.3	7.8–8.2	38º 50′ N, 1º 36′ W
Pétrola lake	2.7–50.3/142*	8.2–8.6	38º 50′ N, 1º 33′ W
Corral Rubio lake	3–10.7	7.6–8.4	38º 50′ N, 1º 27′ W

* After the summer, one of the sections of the lake was almost dry and extremely saline, however the salinity in the main lake was 50.3 g/L.

**Table 2.** Salinity range in which we found active individuals of each new species.

Species	Salinity range (g/L)
*Arcella salobris*	5.3–13
*Arcella euryhalina*	4.3–36 (in the culture at 50 g/L they survived 4 months, with some divisions)
*Galeripora marichusae*	7.3–19.3 (2 cells were kept at 50 g/L and survived for several months without dividing)

### New strains isolated and corresponding morphometric analyses

We isolated four different strains from saline aquatic environments and identified them based on their test morphology: (1) a strain from the *Galeripora discoides* group (González Miguéns et al. 2022), in sediments of the Pétrola lake, that we will describe as *Galeripora marichusae* sp. nov.; (2) a strain resembling *Arcella intermedia*, also from Pétrola lake, that will be described as *Arcella euryhalina* sp. nov.; (3) a strain also resembling *Arcella intermedia* from a saline stream in Salobral de Ocaña, *Arcella salobris* sp. nov.; and (4) a strain from the *Galeripora arenaria* species complex in terrestrial mosses growing on saline soils, in Salobral de Ocaña, *Galeripora halaurula* sp. nov.

**Figure 1.**
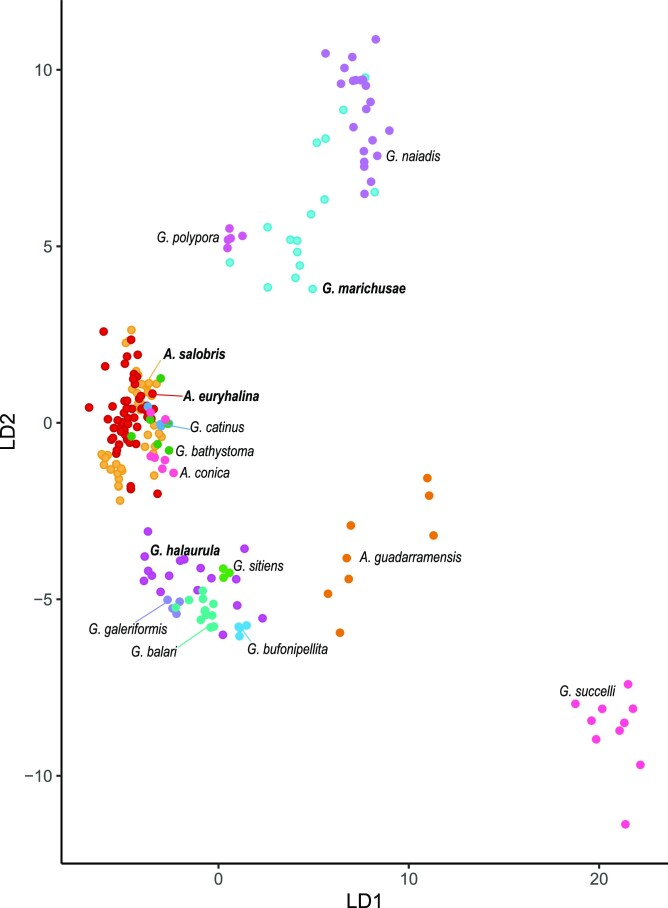
Scatterplot representation of linear discriminant analysis (LDA) based on the morphometric measurements (aperture diameter, test diameter and ratio aperture/test) of the different species. Each color represents a species; in bold letters are the species described in this paper, the rest are from González-Miguéns, Soler-Zamora, Villar-Depablo et al. (2022)

There is some overlap between species morphometry in the PCA and LDA. The LDA was able to correctly assign the specimens with 75.5% accuracy. *Galeripora marichusae* sp. nov. has some overlap with *Galeripora naiadis*, although it is generally smaller; *Galeripora halaurula* sp. nov. with *G. sitiens* and, to a lower extent, *G. balari; Arcella euryhalina* sp. nov. and *Arcella salobris* sp. nov. have some overlapping between them (Fig. 1), but in *Arcella euryhalina* sp. nov. the preapertural area is more clearly delimited than in *Arcella salobris* sp. nov. Another species isolated in Brazil, *A. uspiensis* (according to the measurements in Porfírio-Sousa et al. (2017) and Ribeiro et al. (in preparation)), also has a marked preapertural area, but the lip surrounding the aperture is much thinner.

### Barcoding, phylogenetic analysis and ancestral character reconstruction

Table 3 indicates the Genbank reference numbers for all the different obtained sequences, as well as how many isolates we tried to sequence and how many were successfully sequenced.

**Table 3.** COI sequences obtained for each species. N_i_ indicates the number of tubes with isolated individuals from that species and N_s_ how many of those were successfully barcoded.

Species	Genebank reference	N_i_	N_s_	Habitat	Type locality
*Arcella salobris*	OQ696223	52	2	Sediment (athalassosaline)	39º 59’ N, 3º 37’ W
*Arcella euryhalina*	OQ696225; OQ696226	50	4	Sediment (athalassosaline)	38º 50’ N, 1º 33’ W
*Galeripora marichusae*	OQ696224	15	4	Sediment (athalassosaline)	38º 50’ N, 1º 33’ W
*Galeripora halaurula*	OQ696227	9	6	Soil (brown moss)	39º 59’ N, 3º 37’ W

The monophyly of Arcellidae is recovered with a Bayesian posterior probability (PP) of 1 and a maximum likelihood bootstrap (ML) of 100. Genus *Galeripora* is recovered with PP = 0.62 and ML = 87. However, *Arcella euryhalina*, which has the morphological characteristics of *Arcella*, branches as a sister clade to all *Galeripora* instead of within *Arcella* (PP = 0.98 and ML = 95).

The individuals obtained from the sampled athalassic saline environments appear in different parts of the tree. *Arcella salobris* appears to be genetically closely related to the freshwater species *A. uspiensis*, forming a strongly supported clade (PP = 1, ML = 100). *Arcella euryhalina*, in turn, forms a clade with an environmental sequence (ON651640) from freshwater Lake Oromocto (Canada) (PP = 0.93, ML = 82). *Galeripora marichusae* appears well nested within the aquatic *Galeripora discoides* clade (PP = 0.97, ML = 1), without any clear relationship to the other barcoded species, *G. naiadis, G. bathystoma* or *G. polypora*. Finally, *Galeripora halaurula*, found in terrestrial brown mosses in the area of Salobral de Ocaña, appears within the terrestrial clade of *Galeripora* (PP = 1, ML = 98), grouped with several environmental sequences from brown mosses samples (PP = 1, ML = 100), most of them from coastal areas that receive salt spray; one sequence also came from an inland, non-saline location (González-Miguéns et al. 2023). The closest described sister species would be *G. sitiens* (PP = 1, ML = 82).

**Figure 2.**
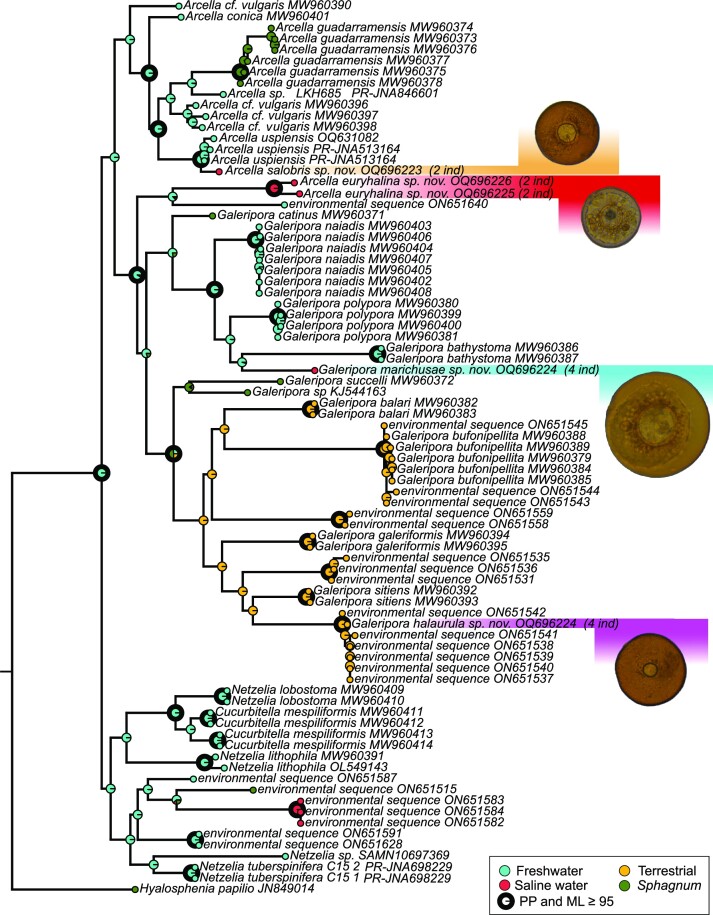
Phylogeny with ancestral character reconstruction of the habitat. Nodes with a support of at least 95 in both maximum likelihood and Bayesian posterior probability appear with an emboldened black circle. The species described in this paper appear highlighted, with their respective image and with a number in brackets indicating how many individuals had that same sequence.

The reconstruction of the ancestral habitat recovered freshwater environments as the most likely ecosystem for the ancestor of the family Arcellidae. The independent ecological transitions through the salinity barrier in Arcellidae can be seen (Fig. 2), corresponding to three of the new species found in this study (see Taxonomic actions and species accounts). There is also one ecological transition in family Netzeliidae corresponding to environmental sequences from subtidal marine sediment samples, obtained by metabarcoding in González-Miguéns et al. (2023). The humidity barrier has only been crossed once, in the *Galeripora arenaria* species complex group, being a monophyletic group of terrestrial environments.

### Growth at different salinities

**Figure 3.**
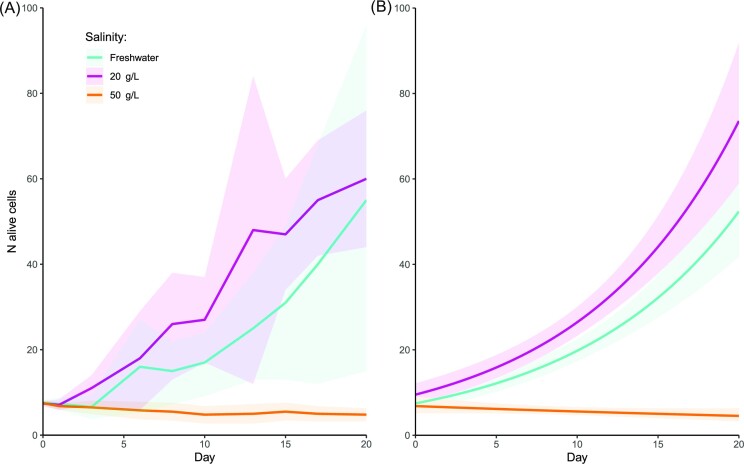
Growth rates of the cultures of *Arcella euryhalina* at different salinities. Each shaded area represents the 95% confidence interval. (**A**) Graph with raw data. (**B**) Graph with the adjusted negative binomial model.

Cultures of the *Arcella euryhalina* specimens from Laguna de Pétrola grew both in freshwater and in water with a salinity of 20 g/L without significant differences (*P* = 0.676), reaching, after 20 days, 55.4 ± 43.6 and 60 ± 16.8 alive individuals, respectively (Fig. 3). Cultures at a salinity of 50 g/L behaved significantly differently from the other treatments (*P* = 1.1 x 10^−13^): we saw little foraging activity and no divisions during the time of the experiment. The number of individuals slowly decreased from the eight initial individuals to 4.8 ± 1 after 20 days. Nevertheless, the source culture at 50 g/L remained alive for more than 4 months after the experiment, and we even saw instances of cell division, even although these events were very uncommon.

### Taxonomic actions and species accounts

We followed an integrative approach based on ecological, molecular and morphological results to make taxonomic decisions on the new mitochondrial lineages obtained in this study. The taxonomic decisions were taken in accordance with the rules and recommendations of the International Code of Zoological Nomenclature (ICZN, 1999), which apply to testate amoebae (Lahr et al. 2012, Adl et al. 2019).

*Family* Arcellidae *Ehrenberg, 1843*

*Genus* Arcella *Ehrenberg, 1830*

**Figure 4.**
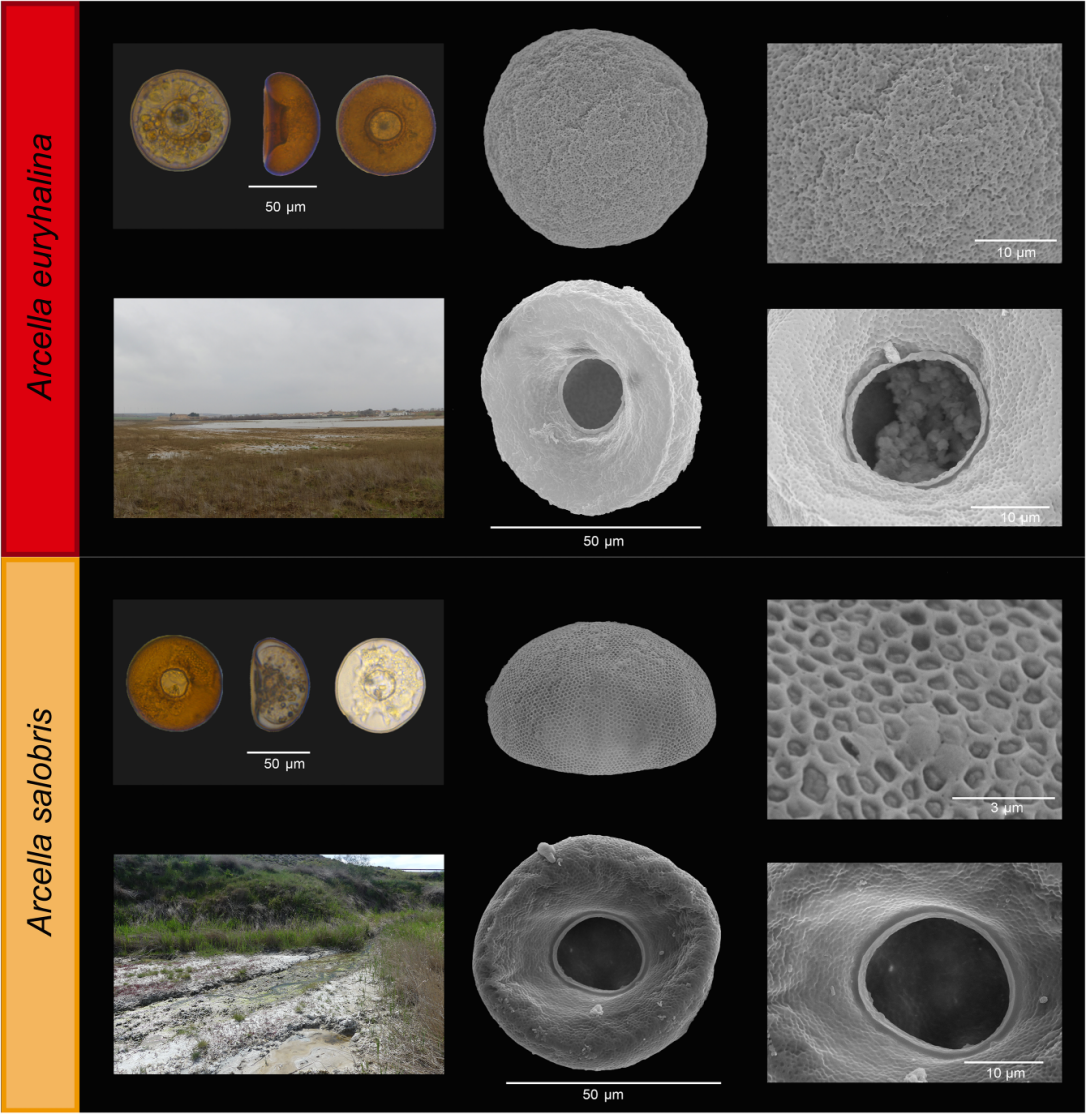
*Arcella euryhalina* and *A. salobris*. Frontal and lateral view on optical microscopy and scanning electron microscopy photographs of the oral and aboral sides of the test, along with details of the test and the aperture. The photograph on the bottom left represents an image of the collection site.

*Arcella euryhalina* sp. nov. (Fig. 4)

**Zoobank registration**: 89237CDC-CF8C-46F9-A7DB-F421B517C7CF

**Holotype**: MA-Amoeba 11267. Royal Botanical Garden, Madrid, Spain.

**Specific diagnosis**: Test diameter of 58–77 µm, average of 68.5 µm (N = 53) and standard deviation (SD) of 4.18 µm. Aperture diameter of 16–26 µm, average of 21.0 ± 2.2 µm (N = 52). Aperture diameter with rim of 19–30 µm, average of 25.5 ± 2.4 µm (N = 49). Height of 30–45 µm, average of 39.7± 4.2 µm (N = 13).

Round test, circular in oral and aboral view, hemispherical in lateral view. Round borders, no ridges or depressions. The aperture is circular and invaginated, surrounded by a thin collar. The borders of the preapertural area are generally clear and well marked. There is a very large amount of cement between the building units of the cell, which covers the majority of the test surface.

**Diagnosis with closely related species:** *Arcella euryhalina* can be diagnosed by its specific mtDNA (COI) sequence. It is morphologically very similar to *A. salobris*, but the preapertural area is generally wider and with a more marked margin in *A. euryhalina*. It also has a thicker cement layer between the building units.

***Terra typica*:**Lake Pétrola, Albacete Province, Castilla La Mancha, Spain (38º 50′ N, 1º 33′ W).

**Habitat:** Aquatic, variable salinity (4 to 36 g/L). In the sediment of an athalassohaline lake. In culture, these organisms grew optimally in pure freshwater, and survived at 50 g/L.

**Derivatio nominis:** The epithet *euryhalina* refers to the fact that is an organism able to live in a wide range of salinities.

*Arcella salobris* sp. nov. (Fig. 4)

**Zoobank registration**: 6C7F58A1-CFB2-4667-B239-D07AEEDCC37C

**Holotype**: MA-Amoeba 11268. Royal Botanical Garden, Madrid, Spain.

**Specific diagnosis**: Test diameter of 70–78 µm, average of 73.9 µm (N = 28) and SD of 1.97 µm. Aperture diameter of 18–27 µm, average of 22.7 ± 1.9 µm (N = 25). Aperture diameter with rim of 22–27 µm, average of 24.5 ± 1.6 µm (N = 6), although the rim was only visible on a few individuals. Height of 37–52 µm, average of 43.5 ± 4.4 µm (N = 7).

Round test with hemispherical shape and round borders. Thin lip surrounding the aperture. The preapertural area is not clearly marked, its margins are generally smooth. Because of this, the preapertural area is often difficult to differentiate in the apertural view. The test is formed by building units surrounded by relatively thick cement. Pores can be observed in the cement.

**Diagnosis with closely related species:** *Arcella salobris* can be diagnosed by its specific mtDNA (COI) sequence and its phylogenetic placement. It is morphologically very similar to *A. euryhalina*, but the preapertural area has smooth borders and is not so clearly visible in *A. salobris. Arcella salobris* also tends to have a more hemispherical shape in the lateral view. It is very similar to both *A. hemisphaerica* and *A. rotundata*, but those species do not have thick cement between their building units, according to the images in Lahr and Lopes (2009), and have not been recorded in athalassohaline environments.

***Terra typica*:** Salobral de Ocaña, Toledo Province, Castilla la Mancha, Spain (39º 59′ N, 3º 37′ W).

**Habitat:** Aquatic, variable salinity (observed as active between 5–13 g/L; given the characteristics of the sampling site, the tolerance range may be wider). In the sediment of an athalassohaline little stream.

**Derivatio nomini:** *salobris* means ‘with salt’; the epithet also refers to the place where they were collected: ‘Salobral de Ocaña’.

*Genus* Galeripora *González-Miguéns, Soler-Zamora, Villar-dePablo, Todorov & Lara*

**Figure 5.**
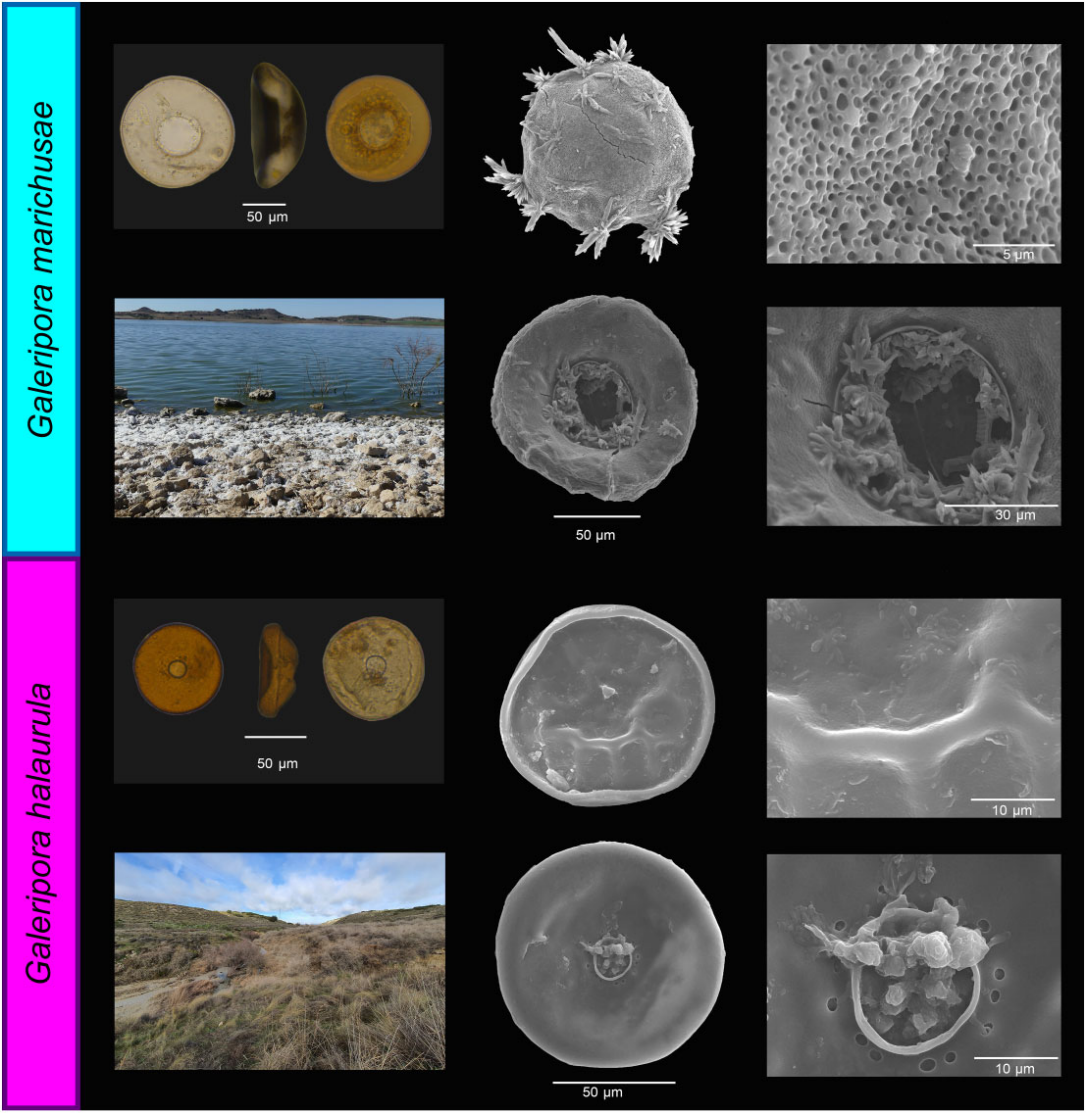
*Galeripora marichusae* and *G. halaurula*. Frontal and lateral view on optical microscopy and scanning electron micrographs of the oral and aboral sides of the test, along with details of the test and the aperture. The projections on the test of *G. marichusae* are salt crystals. The photograph on the bottom left represents an image of the collection site.

*Galeripora marichusae* sp. nov. (Fig. 5)

**Zoobank registration**: 7E1B03D6-7359-4D86-AE67-857B3C74E41A

**Holotype**: MA-Amoeba 11269. Royal Botanical Garden, Madrid, Spain.

**Specific diagnosis**: Test diameter of 103–150 µm, average of 127.3 µm (N = 16) and SD of 12.2 µm. Aperture diameter of 36–57 µm, average of 44.1 ± 5.8 µm (N = 16). Aperture diameter with rim of 43–65 µm, average of 51.4 ± 6.3 µm (N = 14). Height of 23–28 µm, average of 25.39 ± 3.19 µm (N = 2).

Smooth test, without ridges or hollows and with a discoid, flattened shape. Composed of small building units surrounded by an organic cement. The aperture is large and round, invaginated and bordered with a lip, with several small pores surrounding it.

**Diagnosis with closely related species:** *Galeripora marichusae* can be diagnosed by its specific mtDNA (COI) sequence and its phylogenetic placement, as well as its morphometric characteristics. Its flat profile and numerous apertural pores are typical for the *G. discoides* group (González Miguéns et al. 2022). Its diameter is situated between *G. naiadis* and *G. polypora*, although with some degree of overlapping. This is the only species of the group found in athalassohaline environments to date.

***Terra typica*:** Lake Pétrola, Albacete Province, Castilla la Mancha, Spain (38º 50′ N, 1º 33′ W).

**Habitat:** Aquatic, variable salinity (4 to 19 g/L). On the sediment of athalassohaline lakes. In culture, they could survive at a salinity of 50 g/L.

**Derivatio nominis:** *marichusae* is in honour of Maria Jesús Dabad-Moreno, who lives in a village next to Pétrola lake, where this species was found.

*Galeripora halaurula* sp. nov. (Fig. 5)

**Zoobank registration**: 5D639831-3BBC-428B-A6D2-C032D528BA85

**Holotype**: MA-Amoeba 11270. Royal Botanical Garden, Madrid, Spain.

**Specific diagnosis**: Test diameter of 65–98 µm, average of 79.1 µm (N = 17) and SD of 10.5 µm. Aperture diameter of 11–20 µm, average of 14.2 ± 2.1 µm (N = 17). Aperture diameter with rim of 12–22 µm, average of 15.6 ± 2.3 µm (N = 17). Height of 28.11 µm (N = 1).

Shell composed of small building units, but there is frequently a smooth organic matrix that covers them and prevents their observation, particularly in the oral side. On the aboral side the building units give it a rough toad-like texture. There is no cement between them.

The center of the oral side is concave, but with a smooth transition, without a clearly delimited preapertural area. The aperture is small in relation with the diameter of the test; it has a lip and is surrounded by several relatively large pores. In some individuals there were a few other isolated pores on the margins of the oral side. In the lateral view it looks like a trapezoid, but the border protrudes. It resembles a hat. On the aboral side there are some smooth, radial ridges. In most dead individuals the test was flattened.

**Diagnosis with closely related species:** *Galeripora halaurula* can be diagnosed by its specific mtDNA (COI) sequence and its phylogenetic placement. It is morphologically very similar to *Galeripora arenaria, G. sitiens, G. bufonipellita* and *G. balari*.

***Terra typica*:** Salobral de Ocaña, Toledo, Spain (39º 59′ N, 3º 37′ W).

**Habitat:** Terrestrial, growing on aerophilic mosses in dry soil, with salt deposits. Environmental metabarcodes showed its presence in mosses growing on sandy beaches, exposed to the sea breeze. It has also been found, however, away from the sea or any salt source; this suggests that this species is also euryhaline.

**Derivatio nominis:** Halaurula derives from the Greek ‘halo’ for ‘salt’, and ‘aurula’ from Latin for ‘gentle breeze’, referring to the fact that we found this species in mosses in locations where they may be exposed to salt spray or pollution.

## Discussion

### Phylogenetic placement of the isolated organisms

The phylogenetic placement of *Arcella salobris, Galeripora marichusae* and *G. halaurula* are concordant with their morphology according to the phylogeny presented in González-Miguéns, Soler-Zamora, Villar-Depablo et al. (2022). In turn, *Arcella euryhalina* branches as a sister group to the whole genus *Galeripora* but lacks the synapomorphies of the group: pores surrounding the aperture and an organic matrix that covers at least partially the oral side (González-Miguéns, Soler-Zamora, Villar-Depablo et al. 2022). *Arcella euryhalina* has a similar overall shell morphology to *A. salobris* or *A. uspiensis* (Ribeiro et al. (in preparation)) and should be considered as belonging to the genus *Arcella*. Genus *Arcella* then becomes paraphyletic, according to this tree. Family Arcellidae is morphologically very diverse (Deflandre 1928) and still remains underexplored molecularly; incomplete taxon sampling may affect the topology of the actual phylogenetic tree. Therefore, we provisionally keep this taxon within genus *Arcella* until a more complete picture of family Arcellidae is available.

The validity of granting a specific status to *A. salobris* can be discussed. This species is morphologically very similar to *A. uspiensis* (Ribeiro et al. (in preparation)); it is slightly smaller but the dimensions overlap (i.e. shell diameter 64–76 μm in *A. uspiensis* vs. 59.6–77.9 μm in *A. salobris*; aperture diameter 16–22.8 μm in *A. uspiensis* vs. 15–26.4 μm in *A. salobris*). Genetically, both species share 96.2% sequence similarity in the COI fragment analyzed here; this distance overcomes all distances encountered within species in Arcellidae, except for *A. guadarramensis*, which is suspected to also be a species complex (González-Miguéns, Soler-Zamora, Villar-Depablo et al. 2022). While both species are very similar to each other, they diverge deeply in their ecology. Indeed, *A. uspiensis* has been collected from freshwater sediments in Brazil (Ribeiro et al. (in preparation)) and experimental evidence suggests that it dies above 2 g/l NaCl (D. Singer (personal communication)), whereas *A. salobris* was found active at salinities of 5–13 g/L (Table 2). All this suggests important ecological differences, which, together with their genetic distance and their remote respective geographical origins, would indicate that these taxa represent two independent evolutionary units, that is, two different species.

### Inland saline lake populations are euryhaline

Our records would be the highest salinity in which active organisms, not only from family Arcellidae, but also the whole order Arcellinida, have been found. Indeed, these organisms were reported in coastal salt marshes at a maximum salinity of 13 g/L (Charman et al. 2002, Gehrels et al. 2006), but more frequently at salinities below 5 g/L (Golemansky 1998, Gehrels et al. 2006). Our observations are also, to the best of our knowledge, the first records of Arcellinida living in athalassohaline environments.

Inland saline water bodies are characterized by fluctuating parameters, notably salinity, which drops drastically during the rainy season and in some cases can reach saturation after the dry season. Therefore, athalassohaline systems can be expected to host euryhaline species that can cope with the sudden changes. In line, all three aquatic species described here have been found active in salinities which range from a few grams per liter to a salinity comparable with the sea in the case of *A. euryhalina* (Table 2). Our experiments with *A. euryhalina* show that it grows equally well in freshwater and at 20 g/L. Furthermore, this species was capable of long-term survival in a salinity of 50 g/L. Under these conditions, cells were mostly inactive, but did not die and eventually reproduced (although rarely). They are, thus, capable of adapting to a wide range of environmental conditions.

A similar pattern has been seen in other organisms living in athalassohaline lakes. For example, the dinoflagellate *Biecheleria tirezensis*, found in the nearby saline lake of Tirez (Spain), has been cultured in salinites from 2 up to 56 g/L (Raho et al. 2018). Metabarcoding studies have also found some centrohelid species that are present in inland lakes with salinities of 1 g/L and up to 78 g/L (Gerasimova et al. 2020). There are other examples in rotifers (Fontaneto et al. 2006), diatoms (Nakov et al. 2020) or *Aspergillus* (Nazareth and Gonsalves 2014). All these examples show a common feature for organisms in athalassohaline ecosystems; they must have a high tolerance to osmotic stresses through metabolic changes, similar to what is observed in other euryhaline systems, the coastal zones (Lahlou et al. 1969, Nordlie and Haney 1993, Meng et al. 2013). The potential presence of euryhaline Arcellidae in limnoterrestrial systems still needs to be evaluated. More data are needed to assess their distribution across the different ecosystems and to evaluate their realized niche.

### Frequency of ecological transitions across the salinity and humidity barriers

Transitions across the salinity barrier have always been considered infrequent in the evolutionary histories of the different clades, especially of microorganisms (Logares et al. 2009). However, there is no precise definition of what can be called ‘frequent’ or ‘infrequent’, which results in subjective applications of these terms. Moreover, this concept appears even less obvious in taxa where the systematics framework is far from complete and where only a part of the diversity is known, as is the case in most microbial groups.

In many cases there may also be a bias in the environmental sampling. Most reported transitions occur from marine to freshwater environments (Lee and Bell 1999, González-Miguéns, Soler-Zamora, Fernando Useros et al. 2022), with few cases in the opposite direction (Riek 1977). But marine ecosystems also have been better studied, at least for protists, than terrestrial and freshwater ecosystems (Geisen et al. 2017). In fact, Jamy et al. (2022) found equivalent freshwater-to-marine and marine-to-freshwater transition rates in their models across the whole eukaryotic tree based on environmental sequencing data, as well as hundreds of transitions. Within saline environments, marine ecosystems have been studied much more than athalassohaline ones. And within athalassohaline environments there also seems to be a bias towards hypersaline, highly extreme habitats, rather than subhaline or hypohaline lakes.

Furthermore, groups that are traditionally studied by the marine biologists community are hardly studied in freshwater environments. For example, foraminifera are a mainly marine group, but with a few species recorded from freshwater habitats (Siemensma et al. 2017). However, freshwater foraminifera were largely ignored during most of the 20th century (Holzmann et al. 2021). Inversely, the traditionally soil and freshwater group Trebouxiophyceae have been barely studied in the ocean, although it is present (Metz et al. 2019). Drawing conclusions about the frequency or the directionality of the ecological transitions of the salinity barrier is therefore highly challenging, given the limited extension of our knowledge of protist diversity and also the uneven sampling effort between the different environments.

This is also the case in the family Arcellidae, as well as in the whole class Arcellinida, where the great majority of studies focus on freshwater and soil ecosystems, particularly peatlands. A few studies found some halotolerant species in hypohaline coastal lagoons and marshes (Golemansky 1998, Charman et al. 2002, Gehrels et al. 2006). However, they had never been studied in athalassohaline environments. After this study of Arcellidae from these environments, we have found not only new biodiversity (i.e. four new species), but also three independent ecological transitions from freshwater to saline ecosystems. These numbers are likely to increase as more Arcellinida that inhabit athalassohaline lakes will be sampled, especially in hypo and mesohaline systems.

The fluctuating salinity of estuaries and coastal lagoons may have facilitated ecological transitions to colonize, respectively, marine or freshwater systems (Lee and Bell 1999). In athalassohaline ecosystems they may also have played the role of a stepping stone. There, in absence of contact with the sea, colonization events are expected to occur from freshwater to high salinity ecosystems (Beadle 1969, Fontaneto et al. 2006, Bayly and Boxshall 2009).

In contrast to these three ecological transitions across the salinity barrier in Arcellidae, the phylogeny of the family reveals a single transition towards terrestrial environments, even although terrestrial environments have been historically more sampled than saline ones in this family. This transition occurred at the emergence of the *Galeripora arenaria* species complex. This clade is morphologically very homogeneous and obtains maximum support in molecular phylogenies (González-Miguéns, Soler-Zamora, Villar-Depablo et al. 2022). Wet bryophytes such as *Sphagnum* mosses, which is a highly humid, sometimes subaquatic environment (Booth 2008), may have played a key role as a transition environment in the preadaptation of freshwater species to soil ecosystems. The position of *Galeripora succelli* and *Galeripora* sp. KJ544163, both isolated in *Sphagnum*, as sister group to the whole *G. arenaria* species complex (Fig. 2), corroborates this hypothesis.

Terrestrializations are also rarer than salinity transitions in other groups of eukaryotes: in Decapoda, Davis et al. (2022) report numerous marine-to-freshwater transitions in several clades, but only four transitions to the terrestrial environment (one in Anomura and three in Brachyura). This pattern repeats in molluscs (Aristide and Fernández 2022), annelids (Rousset et al. 2008) and nematodes (Holterman et al. 2019). Several groups that actually crossed the salinity barrier were unsuccessful in settling terrestrial environments, like Gastrotricha (Kolicka et al. 2020), Tintinnid ciliates (He et al. 2022) or Caridean shrimps (Davis et al. 2018). In summary, when extending to all eukaryotes (microbial or not), the humidity barrier could play a role at least as important as the salinity barrier in the structuration of biodiversity worldwide.

### An hypothesis to explain the absence of arcellinida in the sea

#### The paradox of the absence of Arcellinida in marine systems

The fact that multiple lineages of Arcellidae and Netzeliidae have been able to adapt to salinities comparable with seawater (Fig. 2) suggests that they should have been physiologically able to colonize oceans. However, the records of Arcellidae found alive in the sea are scarce, and are mainly limited to brackish coastal marshes with low salinities (Golemansky 1998, Charman et al. 2002, Gehrels et al. 2006). This suggests that they are possibly absent from marine systems, despite the fact that abiotic parameters should not prevent their presence in these environments. They can cross the salinity barrier, but not colonize the sea, or more saline coastal lagoons that are connected to the sea. A similar pattern can be found in Anostraca (Eng et al. 1990, Mura 1999) and the green alga *Dunaliella* (Assunção et al. 2012, González et al. 2019): they are typical inhabitants of athalassohaline ecosystems, but are absent from marine environments. Because salinity is not the barrier preventing these clades from conquering the ocean, or at least not on its own, it can be hypothesized that biotic parameters, such as predation and/or competition, may be playing a role.

#### Ecological transitions are favored under low biotic pressure

Would-be colonizers are under a competitive disadvantage against well-adapted local species, hindering their possibilities of colonizing the new environment (Vermeij and Dudley 2000). In fishes, competition with extant species seems to be a factor limiting diversification after ecological transitions (Betancur-R. et al. 2012). In oceanic islands, where many predators are absent and interspecific competition is reduced, many species colonize new niches from which they are usually excluded in the continent. For example, the four only known aquatic-to-terrestrial transitions in truncatellid gastropods all occurred on oceanic islands (Rosenberg 1996, Vermeij and Dudley 2000). Darwin's finches (Grant and Grant 1982) and *Anolis* lizards’ convergent evolution (Kolbe et al. 2011) are also typical examples where colonists find empty niches in islands and, in the end, speciate. These lower biotic pressures may have facilitated, or allowed, the survival of ecological transitioners that otherwise would have been outcompeted. Lakes have been considered as analogues of islands in many biogeographical studies (Browne 1981, MacDonald et al. 2018). Thus, it could be expected that interspecific competition and predation in athalassohaline lakes is lower than in the sea, which would be analogous to ‘the mainland’. This could have facilitated the adaptation to this niche of the colonizers coming from freshwater environments. Because sampling efforts have been more focused on marine-to-freshwater transitions, our perception of how difficult it is to cross the salinity barrier may be biased. Further sampling of these athalassohaline ecosystems may reveal many more transitions across this barrier.

#### What could be excluding Arcellinida from the marine environment?

Predation and/or competition that could prevent Arcellidae from settling marine sediments may come from many benthic shelled protists with similar lifestyles like Gromiids, Euglyphids, small Metazoa or possibly Foraminiferans. Foraminiferans are a mainly marine group, although there are also a soil and a few freshwater species (Holzmann et al. 2021) and some reported in athalassohaline lakes (Plaziat 1991). Foraminifera and Arcellinida tend to overlap in coastal lagoons and other brackish environments, but Foraminifera usually dominate when the salinity is higher, and vice versa (Charman et al. 2002, Vázquez Riveiros et al. 2007, van Hengstum et al. 2008). They could be a candidate group that could exclude Arcellidae from marine sediments (Whittle et al. 2018), although the putative competition should be empirically tested. Mesocosm experiments in seawater tanks inoculated with salt-tolerant Arcellidae and benthic Foraminiferans can be instrumental in testing this hypothesis.

## Conclusion

Here we report the presence of Arcellidae, a mainly freshwater and terrestrial group, in athalassohaline environments, living and thriving at salinities as high as seawater and facing strong salinity fluctuations. These new species represent three independent freshwater-to-saltwater transitions, as opposed to one single aquatic-to-terrestrial transition within Arcellidae. Most probably, transitions across the salinity barrier are likely to occur frequently, as the new species we describe here have been obtained from a single region (Spain); further diversity will certainly be revealed when the sampled regions are expanded. This opens the door to further research exploring the diversity of Arcellidae and other protists in athalassohaline environments, the evolutionary history of these halotolerant or halophilic groups, their ecology and the physiological, cytological and molecular basis of salinity tolerance in the group.

Arcellidae are a primarily freshwater group with a high potential for colonizing saline systems, and less so for soil. However, adapting to a new ecosystem type also requires overcoming biotic constraints such as predation or competition. These constraints, and not only the abiotic ones, need to be taken into account when studying ecological transitions in all organisms, microbial and multicellular alike.

## Supplementary Material

fiad076_Supplemental_Files
